# Corrigendum to “Systematic comparisons between Lyme disease and post-treatment Lyme disease syndrome in the U.S. with administrative claims data”

**DOI:** 10.1016/j.ebiom.2023.104652

**Published:** 2023-06-19

**Authors:** Ming Kei Chung, Mariaelena Caboni, Philip Strandwitz, Anthony D'Onofrio, Kim Lewis, Chirag J. Patel

**Affiliations:** a Harvard Medical School, Boston, MA, USA; b Northeastern University, Boston, MA, USA

Some researchers hypothesize that PTLDS might be related to a persistent but difficult to detect infection. The authors wish to improve the clarity of a statement in the background section of the summary. The updated background section is presented below:

Summary

**Background** Post-treatment Lyme disease syndrome (PTLDS) is used to describe Lyme disease patients who have completed antibiotic therapy but then experience persisting symptoms of pain, fatigue, or cognitive impairment.

